# ST2 deletion enhances innate and acquired immunity to murine mammary carcinoma

**DOI:** 10.1002/eji.201141417

**Published:** 2011-04-12

**Authors:** Ivan Jovanovic, Gordana Radosavljevic, Maja Mitrovic, Vanda Lisnic Juranic, Andrew N J McKenzie, Nebojsa Arsenijevic, Stipan Jonjic, Miodrag L Lukic

**Affiliations:** 1 Center for Molecular Medicine and Stem Cell Research, Faculty of Medicine, University of KragujevacKragujevac, Serbia; 2 Departments of Histology and Embryology, Faculty of Medicine, University of RijekaRijeka, Croatia; 3 Medical Research Council Laboratory of Molecular BiologyCambridge, UK

**Keywords:** Cytotoxicity, 4T1 mouse breast cancer, NK cells, ST2, Th1/Th2 cells

## Abstract

ST2 is a member of the IL-1 receptor family and IL-33 was recently identified as its natural ligand. The IL-33/ST2 pathway regulates Th1/Th2 immune responses in autoimmune and inflammatory conditions, but the role of ST2 signaling in tumor growth and metastasis has not been investigated. We aimed to investigate whether ST2 gene deletion affects tumor appearance, growth, and metastasis, and antitumor immunity in an experimental metastatic breast cancer model. Deletion of ST2 in BALB/c mice bearing mammary carcinoma attenuated tumor growth and metastasis, which was accompanied by increased serum levels of IL-17, IFN-γ, and TNF-α and decreased IL-4. Tumor-bearing ST2^−/−^ mice had significantly higher percentages of activated CD27^high^CD11b^high^ NK cells, CD69^+^ and KLRG^−^ NK cells and higher cytotoxic activity of splenocytes, NK cells, and CD8^+^ T cells in vitro. A significantly higher number of NK cells expressing IFN-γ were found in ST2^−/−^ mice compared with WT recipients. In vivo depletion of CD8^+^ or NK cells revealed a key role for NK cells in enhanced antitumor immunity in ST2^−/−^ mice. We report for the first time that suppressed breast cancer progression and metastasis in mice lacking ST2 corresponds mainly with enhanced cytotoxic activity of NK cells, and increased systemic Th1/Th17 cytokines.

## Introduction

The *ST2* gene, also called T1, DER-4 and Fit-1, is a member of the interleukin-1 receptor (IL-1R) family that was originally identified in oncogene- or serum-stimulated fibroblasts 1, 2. Differential mRNA processing within the *ST2* gene generates three isoforms: a soluble form (sST2), a membrane-anchored form (ST2L), and a variant ST2 (ST2V) which is localized on the plasma membrane and predominantly expressed in the stomach, small intestine, and colon 3–5. Soluble ST2 is found in embryonic tissues, and is secreted by macrophages, Th2 cells, and fibroblasts 1–5. ST2L is an orphan receptor expressed on a variety of cell types including mast cells, basophils, eosinophils, dendritic cells, macrophages, Th2 cells, and NK and iNKT cells 3–15. A member of the IL-1 family, IL-33, previously known as a nuclear factor in high endothelial venules, has been recently described as a natural ligand for ST2L 16. IL-33 is expressed in multiple tissues and has been shown to induce the secretion of both proinflammatory and anti-inflammatory cytokines from mast cells, eosinophils, and Th2 lymphocytes 3, 17. IL-33 is a potent activator of Th2 cells and has a dual role in disease 3, 16. IL-33/ST2 signaling has a protective role against parasites, in atherosclerosis, and obesity, but it can enhance Th2 and mast cell-mediated diseases such as asthma and anaphylaxis. The role of IL-33/ST2 signaling in antitumor immunity is unknown.

Breast cancer is one of the leading causes of cancer deaths among women worldwide 18. The initiation of breast cancer might be caused by a combination of oncogenic mutations that promote genetic instability and accelerated cellular proliferation 19. The major cause of breast cancer deaths is the development of distant organ metastasis in lungs, bones, liver, and brain 20. In breast cancer, antitumor immune responses are neither potent nor effective 21.

Th1 and Th2 cells have important immunoregulatory roles in cancer development 22. Data from numerous reports suggest that Th1-type antitumor immunity provides a greater therapeutic index and promotes durable antitumor CTL responses that are associated with tumor regression. Although Th2-type cytokines downregulate antitumor immunity, they can promote the recruitment of tumoricidal eosinophils and macrophages into the tumor microenvironment 23–26. ST2L is a stable and selective marker of both murine and human Th2 cells, but not Th1 cells 3, 5, 8, 15, 27–29 and ST2L signaling enhances Th2 effector functions 3, 5, 8, 28–30. Abs against ST2, ST2-Fc fusion proteins or use of ST2-deficient mice in various pathological conditions revealed that lack of IL-33/ST2 signaling favors the expansion of Th1/Th17 cells and inhibits Th2 cell-mediated immune responses 3, 5, 28, 31. Activity of natural killer (NK) cells is the major mechanism of innate immunity against tumors 32. NK cells lyse tumor cells without prior sensitization and represent the first line of defense against tumors and cancer metastasis 32. Recently, the ST2L molecule has been shown to be constitutively expressed on murine and human NK and iNKT cells 9, 10.

In this study, using the 4T1 metastatic breast cancer model in ST2-deficient BALB/c mice, we aimed to investigate whether the lack of ST2 signaling affects tumor growth and metastasis and mechanisms of antitumor immunity.

## Results

### ST2-defficient mice show delayed mammary tumor appearance, slower tumor growth, and progression

The 4T1 metastatic breast cancer model is a syngeneic xenograft model in which the 4T1 mouse mammary tumor cell line (5×10^4^ cells) is introduced orthotopically into the mammary fat pad of WT or ST2^−/−^ BALB/c mice by injection. When introduced orthotopically, the 4T1 line grows rapidly at the primary site and forms metastases in lungs, liver, bone, and brain, which makes this an excellent model for the study of metastatic progression of breast cancer in humans. As this model is syngeneic in BALB/c mice, it can be used to study the antitumor immune mechanisms in tumor growth and metastasis 33.

**Figure 1:**
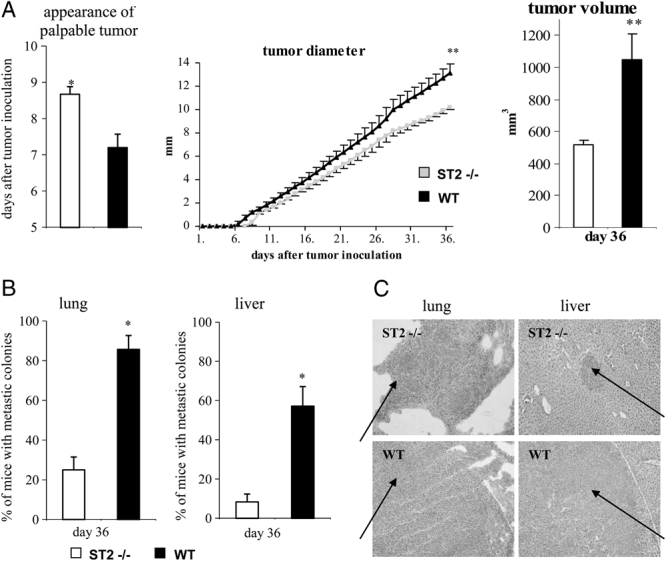
ST2 deletion attenuates tumor growth and metastasis. (A) WT (*n*=7, black bars) and ST2^−/−^ mice (*n*=12, white bars) were inoculated with 5×10^4^ 4T1 breast tumor cells. Palpable tumors were monitored for 36 days. Black line, WT mice; grey line, ST2^−/−^ mice. Tumor diameters were measured every day. Tumor volumes were determined on day 36 from the inoculation of 4T1 tumor cells. Data are presented as mean+SEM from two experiments. Statistical significance was tested by Mann–Whitney Rank Sum test or Student's unpaired *t*-test, where appropriate (*p<0.05; ^**^p<0.01). (B) Paraffin-embedded lung and liver tissues were stained with hematoxylin and eosin (H&E) and examined by light microscopy for the number and size of metastatic colonies. H&E-stained sections (4 μm) from at least three different levels were examined. Data are presented as mean+SEM from two experiments. Statistical significance was tested by Mann–Whitney Rank Sum test or Student's unpaired *t*-test, where appropriate (*p<0.05). (C) Light-microscopic pictures (magnification, 10×) through pulmonary and liver tissue sections showing metastatic colonies (arrows).

After the injection of 4T1 tumor cell line, mammary tumor growth was measured daily and tumor volumes assessed at day 36. As shown in Fig. 1A, tumor appearance was significantly delayed in ST2^−/−^ compared with WT mice. Mean value of time period in days from inoculation of tumor cells to the appearance of palpable primary tumor in ST2^−/−^ mice was significantly longer compared with WT mice (mean±SEM: 8.67 days±0.21 versus 7.20 days±0.37, *p*<0.05). On day 36, the mean value of primary tumor diameter in WT mice was significantly higher than in ST2^−/−^ mice (13.16 mm±0.79 versus 10.18 mm±0.23, *p*<0.01). Similarly, primary tumor volume was significantly higher in WT mice (1047.24 mm^3^±159.48 versus 517.31 mm^3^±26.66, *p*<0.01). Systemic tumor cell dissemination was determined by microscopic evaluation of parenchymal tissues. Six out of seven WT mice (86%) developed numerous lung metastatic colonies, whereas only 3 out of 12 ST2^−/−^ mice (25%) developed lung metastasis (*p*<0.01) as shown in Fig. 1B. The incidence of liver metastasis was also significantly lower in ST2^−/−^ mice (1/12; 8%) compared with WT mice (4/7; 57%, *p*<0.05) (Fig. 1B). Brain metastasis was not found in WT or ST2^−/−^ mice at this time point. In addition to the marked difference in the number of metastatic colonies in lungs and livers, the significant difference in the size of metastatic colonies was also observed, being evidently and significantly smaller in ST2^−/−^ mice compared with WT animals (Fig. 1B and C).

### Cellular makeup of local lymph nodes and spleens in ST2^−/−^ and WT mice after tumor challenge

**Figure 2:**
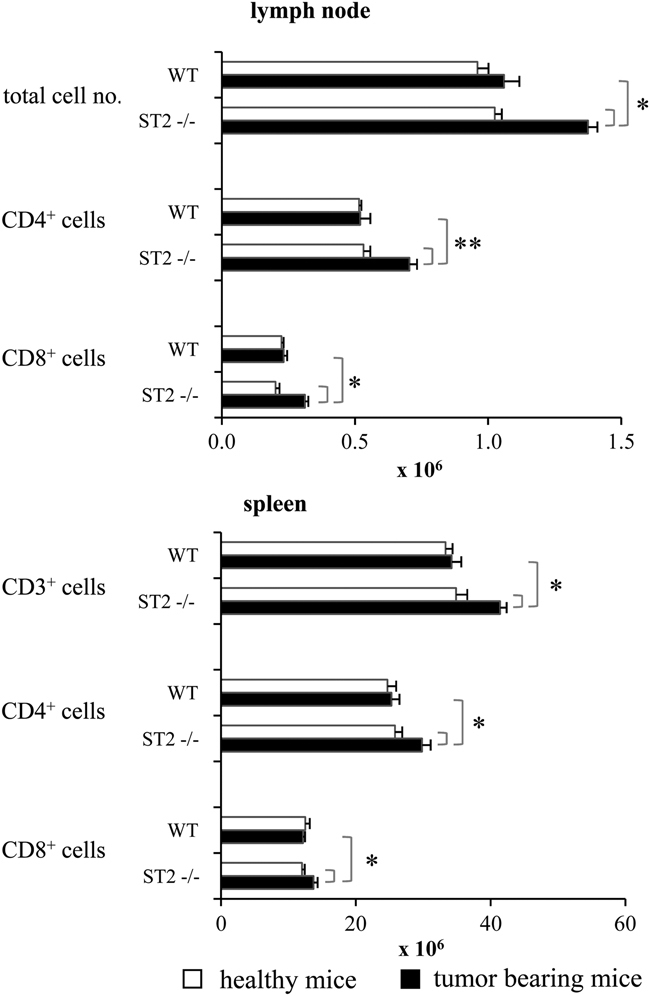
Flow cytometric analysis of the local lymph node and spleen T cells. Total cell number of local lymph nodes and spleens were determined in healthy and tumor-bearing WT and ST2^−/−^ mice on day 13 after inoculation of 4T1 tumor cells. Percentages and absolute numbers of CD3^+^, CD4^+^, and CD8^+^ cells were determined by staining local lymph node or spleen cell suspensions with fluorochrome-labeled Abs and analyzed by a FACSAria flow cytometer (BD Biosciences). Mononuclear cells were gated on by size and granularity on FSC/SSC. Data are presented as mean+SEM of two separate experiments, each carried out with four mice per group. Statistical significance was tested by Student's *t*-test (*p<0.05; ^**^p<0.01).

Tumor cell inoculation led to an increase in cellularity of local lymph nodes in both groups of mice at day 13. Absolute numbers of CD3^+^ cells in the spleens were also increased, but reached statistical significance only in ST2^−/−^ mice (*p*<0.05; Fig. 2). Similarly, percentages (data not shown) and absolute numbers of CD4^+^ and CD8^+^ T cells were significantly increased in local lymph nodes and spleens in ST2^−/−^ mice compared with tumor-bearing WT mice (*p*<0.05; Fig. 2). On the contrary, absolute numbers of CD19^+^ cells were markedly increased in both local lymph nodes and spleens of tumor bearing WT mice compared with ST2^−/−^ recipients (data not shown). We found no difference in the absolute numbers of F4/80^+^ macrophages between ST2^−/−^ and WT animals, prior to and 13 days after tumor challenge (data not shown).

### Increased serum levels of Th1/Th17 cytokines after tumor inoculation in ST2^−/−^ mice

**Figure 3:**
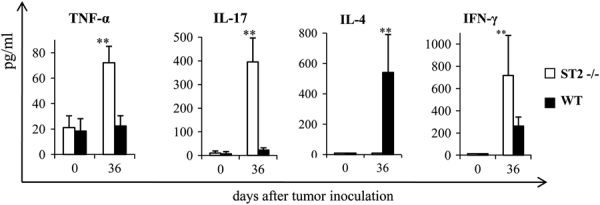
ST2-deficiency enhances systemic Th1/Th17 cytokine levels in mice with established tumors. Serum levels of IL-17, IFN-γ, IL-4, and TNF-α in healthy and tumor-bearing WT and ST2^−/−^ mice were determined by ELISA at day 36 after inoculation of 4T1 tumor cells. Data are presented as mean+SD from at least five mice per group. Statistical significance was tested by Student's *t*-test (*p<0.05;^**^p<0.01).

We measured Th1, Th17, and Th2 cytokine levels in the sera of WT and ST2^−/−^ mice prior to and at day 36 after tumor inoculation. Serum IL-17, IFN-γ, and IL-4 levels were not detectable and serum TNF-α level was low in healthy mice in both groups, i.e. before tumor inoculation. At day 36, tumor inoculation led to a significant increase in serum IL-17, IFN-γ, and TNF-α levels in ST2^−/−^ mice compared with WT animals (*p*<0.05). On the contrary, a significant increase in serum IL-4 levels was observed only in tumor-bearing WT mice (*p*<0.01; Fig. 3).

### ST2-deficient mice have lower number of NK cells, but higher number of IFN-γ^+^ NK cells in spleens

**Figure 4:**
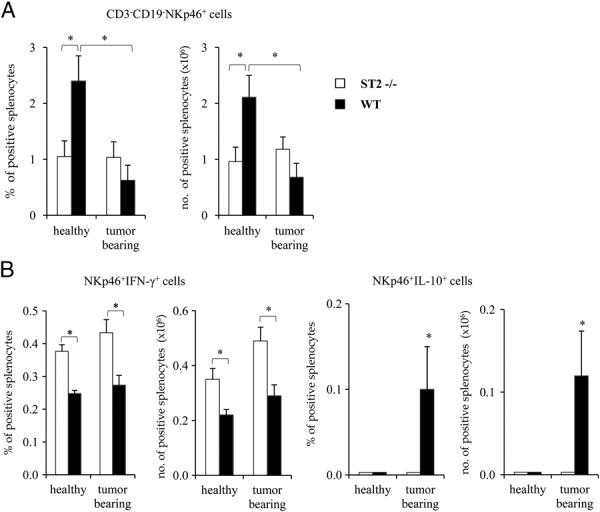
Flow cytometric analysis of NK cell surface markers and intracellular IFN-γ/IL-10 in spleens of tumor bearing ST2^−/−^ and WT mice. (A) The percentages and absolute numbers of CD3^−^CD19^−^NKp46^+^ cells in spleens of healthy and tumor-bearing WT and ST2^−/−^ mice were determined on day 13 after tumor inoculation by using fluorochrome-labeled Abs and analyzed on a FACS Aria. Mononuclear cells were gated on by size and granularity on FSC/SSC. Results are presented as mean+SEM from eight mice per group (*p<0.05). (B) Following surface staining for NKp46 cell marker, splenic cells were permeabilized and intracellular staining of IFN-γ (12 mice per group) and IL-10 (four mice per group) in WT and ST2^−/−^ mice, prior to and on day 13 after tumor inoculation were performed. Data represent mean+SEM from two separate experiments. Statistical significance was determined by Student's *t*-test (*p<0.05).

In order to dissect out the contribution of NK cells in enhanced antitumor activity in ST2^−/−^ mice, we enumerated CD3^−^CD19^−^NKp46^+^ cells. Interestingly, number of these cells in the spleen was lower in tumor naive ST2^−/−^ mice in comparison with WT mice (Fig. 4; *p*<0.05). We also assessed the frequency and cell number of NKp46^+^ cells producing IFN-γ or IL-10 in naïve mice and following tumor challenge. The total number of IFN-γ-producing NK cells was markedly higher in ST2^−/−^ mice prior to and following tumor challenge (*p*<0.05). IL-10-producing NK cells were detected only in WT mice after tumor inoculation (*p*<0.05).

### Lack of ST2 signaling is associated with higher antitumor cytotoxicity

**Figure 5:**
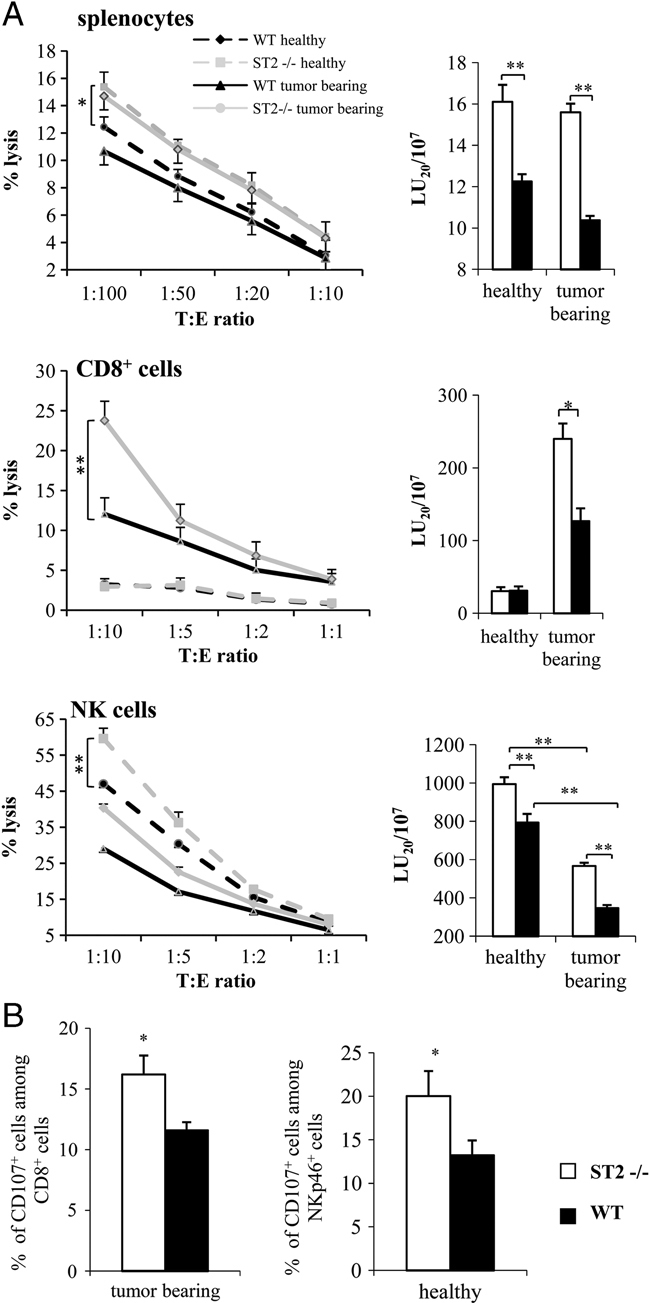
Cytotoxic activity of total splenocytes, NK cells, and CD8^+^ T cells. (A) The cytotoxic activity of effector cell populations was tested in a 4-h MTT assay against 4T1 cell targets, at four different *T*:*E* ratios at day 13. The data are presented as mean percentages of specific cytotoxicity and LU_20_/10^7^ NK cells, which was calculated on the basis of mean percentages of killing in four different *T*:*E* ratios and percentages of effector cells. Data are means+SEM of two individual experiments, each carried out with three mice per group. Statistical significance was tested by Student's *t*-test (*p<0.05; ^**^p<0.01). (B) Flow cytometric analysis of CD107a expression on NKp46^+^ and CD8^+^ cells. Data are means+SEM of two individual experiments, each carried out with three mice per group. Statistical significance was tested by Student's *t*-test (*p<0.05).

ST2 deficiency led to enhanced cytotoxic activity of splenocytes at the target–effector (*T*:*E*) ratios of 1:50 and 1:100 in both healthy and tumor-bearing mice (Fig. 5A). Interestingly, there were no differences in cytotoxic activity of splenocytes in healthy and tumor-bearing ST2^−/−^ mice (evaluated by lytic units), but it was always significantly higher in ST2^−/−^ compared with WT mice (*p*<0.01).

In order to define the effector cells responsible for enhanced cytotoxic capacity of splenocytes, we isolated CD49b^+^ NK cells and tested their antitumor cytotoxicity. The data (Fig. 5) clearly demonstrated higher cytotoxicity of NK cells derived from spleens of healthy ST2-deficient mice when compared with WT mice (*p*<0.01). Interestingly, NK cell activity of the splenic CD49b^+^ cells was decreased in both tumor-bearing groups, but remained significantly higher in ST2^−/−^ mice (*p*<0.01).

We next tested cytotoxic activity of CD8^+^ T cells against tumor cells. As shown in Fig. 5, there was a significant increase in CD8^+^ T cells mediated cytotoxicity after tumor inoculation which was higher in ST2^−/−^ mice in comparison with WT mice (*p*<0.05). In addition, activated NK and T cytotoxic cells (CD107a^+^) were more frequent in ST2^−/−^ mice (Fig. 5B). Similar results were obtained when cytotoxicity and CD107a expression were evaluated using pooled lymph node cells (data not shown). Finally, we found that adherent cells derived from spleen were not responsible for the enhanced cytotoxic capacity of splenocytes derived from ST2^−/−^ mice (data not shown).

### ST2-deficient mice have a higher percentage of active, cytotoxic NK cells with faster turnover

**Figure 6:**
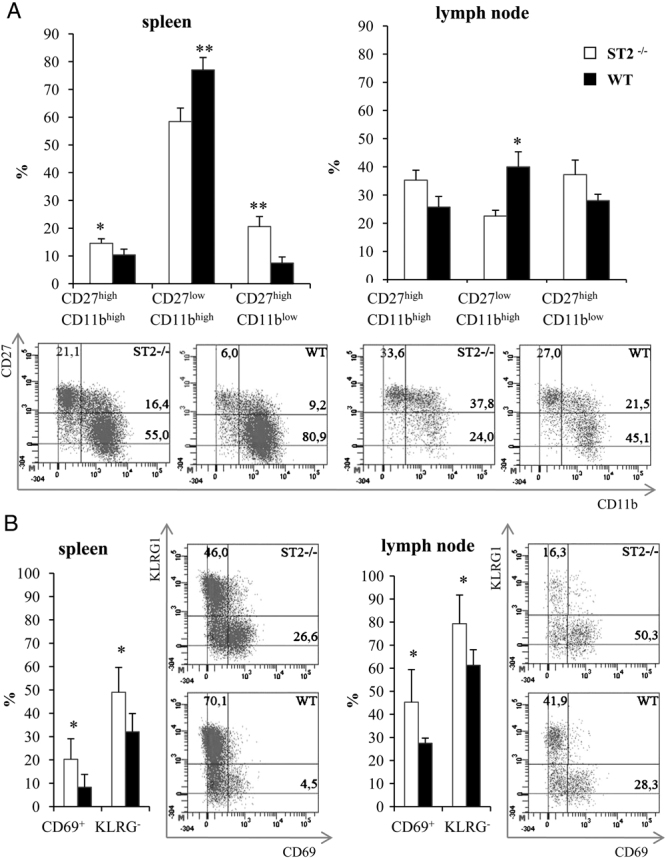
Phenotype of NK cells derived from spleen and local lymph nodes of ST2^−/−^ and WT mice. (A) The percentages and absolute numbers of activated CD27^high^CD11b^high^ NK cells, as well as immature CD27^high^ CD11b^low^ NK cells and mature CD27^low^ CD11b^high^ NK cells in the spleen and local lymph nodes of healthy and tumor-bearing WT and ST2^−/−^ mice at day 13 after tumor inoculation were determined by flow cytometry. Representative dot plots defining CD27^high^/CD27^low^ and CD11b^+/−^CD3^−^CD19^−^NKp46^+^ cells are shown. (B) The percentages and absolute numbers of CD69^+^ and KLRG1^−^ NK cells in the spleens and local lymph nodes of healthy and tumor-bearing WT and ST2^−/−^ mice at day 13 after tumor inoculation were determined by flow cytometry. Representative flow cytometry dot plots show percentages of CD69^+^ and KLRG^−^ CD3^−^CD19^−^NKp46^+^ cells in spleens and local lymph nodes from ST2^−/−^ and WT mice. Data are mean+SD from four mice per group. Statistical significance was determined by Student's *t*-test (*p*<0.05; ^**^p<0.01).

As NK cell cytotoxicity appears to be an important contributor to antitumor activity in experimental mammary carcinoma, we analyzed NK cells for their constitutive phenotype. The data shown in Fig. 6A demonstrate that spleens of ST2-deficient mice had higher percentages of activated, CD27^high^CD11b^high^ NK cells (*p*<0.05) known to be cytokine producing, cytotoxic, and tumoricidal, as well as immature CD27^high^ CD11b^low^ NK cells (*p*<0.01) and a lower percentage of mature CD27^low^ CD11b^high^ NK cells (*p*<0.01). The same trend in NK cell maturation dynamics was observed in local lymph nodes. We also found higher percentages of CD69^+^ and killer cell lectin-like receptor G1 (KLRG1)^−^ NK cells in both spleen and local lymph nodes of ST2^−/−^ mice, compared with WT mice (*p*<0.05; Fig. 6B); the findings support an activated state of NK cells.

### Depletion of NK cells but not CD8^+^ cells accelerates tumor growth in ST2^−/−^ mice

**Figure 7:**
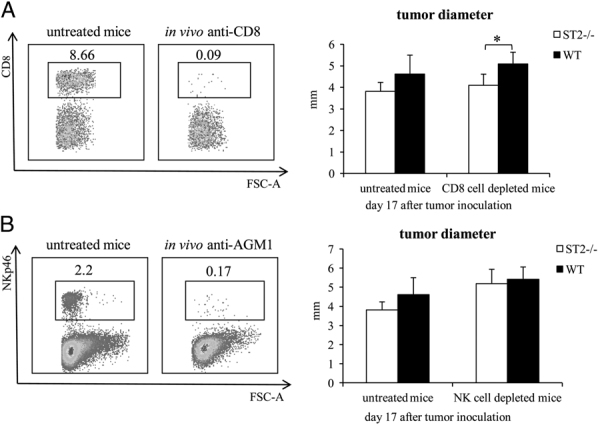
Tumor growth after in vivo depletion of CD8^+^ and NK cells. (A) Mice were injected with 5×10^4^ 4T1 tumor cells and primary tumor growth was evaluated by measuring tumor diameters on day 17 after tumor inoculation in CD8^+^ cell-depleted WT and ST2^−/−^ mice. Representative flow cytometry dot plot shows the percentages of CD8^+^ cells after in vivo administration of anti-CD8 mAb (YTS 169.4, left panel). Data are mean+SD from four mice per group. Statistical significance was determined by Student's *t*-test (*p<0.05). (B) Mice were injected with 5×10^4^ 4T1 tumor cells and primary tumor growth was evaluated by measuring tumor diameters on day 17 after tumor inoculation in NK-depleted WT and ST2^−/−^ mice. Representative flow cytometry dot plot shows the percentages of NK cells after in vivo administration of antiasialo GM1 mAb (left panel). Data are mean+SD from four mice per group.

To evaluate the relative contribution of CD8^+^ cells and NK cells in the enhanced antitumor immunity in ST2^−/−^ mice in vivo, we depleted either one or the other cell population, following tumor challenge in ST2^−/−^ and WT animals. CD8^+^ lymphocytes were depleted by administration of an anti-CD8 mAb on days −1 and +5 of the experiment. Mice were inoculated with 5×10^4^ 4T1 tumor cells and followed for tumor growth (Fig. 7A). Such treatment resulted in a significant reduction of CD8^+^ cells in spleens as shown in Fig. 7A. After in vivo depletion of CD8^+^ cells tumor growth remained slower in ST2^−/−^ mice compared with WT animals, similar to that which was observed in intact animals (Fig. 7A). We next depleted NK cells by in vivo administration of an antiasialo-GM1 Ab as shown in Fig. 7B. When NK cells were depleted in vivo, tumor growth was accelerated in both groups of mice, and the difference of the tumor growth in ST2^−/−^ compared with WT mice could no longer be observed (Fig. 7B). The obtained results indicate a key role for NK cells in the enhanced antitumor immunity in ST2^−/−^ mice.

## Discussion

In the present study, we examined whether the lack of IL-33/ST2 signaling affects antitumor immunity in an experimental murine model of mammary carcinoma. For the first time, we showed the delayed appearance of palpable primary tumor in ST2^−/−^ recipients as well as slower tumor growth. ST2 deletion also inhibited the formation and growth of metastasis. By day 36, the incidence of spontaneous lung and liver metastasis was significantly lower in ST2^−/−^ mice, compared with WT mice. Furthermore, the number and size of metastatic nodules were evidently smaller in ST2^−/−^ recipients (Fig. 1).

Antitumor immunity is mediated by the innate and adaptive immune system 21–24, 32. The important role of T cells in antitumor immunity is well established 22–24. CD4^+^ Th cells play an important role in the regulation of the CD8^+^ T-cell-mediated immune surveillance 23, 24, 26. Th cell polarization influences antitumor activities in a dual manner. Th1 cells preferentially activate cellular immunity (by producing IFN-γ and IL-2) and Th2 cells suppress cellular immunity eliciting humoral immunity (by IL-4, IL-5 IL-10, and IL-13 production; 23, 25, 26, 34). Th1-mediated antitumor immune response is followed by potent stimulation of T-cell cytotoxic activity 22–24, 35. On the contrary, Th2 polarization results in the production of growth factors and cytokines that support tumor growth and metastasis 25. Several studies, using antiST2 mAbs, ST2-Fc fusion proteins or ST2-deficient mice demonstrated that the deletion of IL-33/ST2 signaling favors the expansion of Th1/Th17 cells and have inhibitory effect on Th2-associated immune response in various inflammatory and autoimmune disorders 3, 5, 28, 31, 36, 37.

In our study, the analysis of serum cytokines revealed higher expression of IL-17, IFN-γ, and TNF-α in ST2^−/−^ mice challenged with 4T1 breast cancer cells (Fig. 3). On the contrary, serum IL-4 level was significantly higher in WT mice. Thus, preferential activation of Th1 cells may be important for enhanced antitumor immunity in ST2-deficient mice.

ST2 deletion enhances lymphopoiesis in local lymph node and spleen after tumor inoculation and increases the total number of CD4^+^ and CD8^+^ T cells (Fig. 2). Furthermore, we found significant increase of CD8^+^ T-cell-mediated cytotoxicity in vitro after tumor inoculation in ST2^−/−^ mice in comparison with WT mice.

This hints at a role of IL-33/ST2 signaling in the Th2 polarization of immune response in the breast cancer model. In line with our study, Sweet et al. 38 showed that, after LPS challenge, levels of proinflammatory cytokines were elevated in the sera from mice treated with anti-ST2 Ab compared with the control group, probably by blocking of the Th2 response.

In addition, we examined the cytotoxic capacity of splenocytes and found that ST2^−/−^ splenocytes had enhanced both constitutive and cytotoxic activity after tumor cell inoculation, when compared with WT mice (Fig. 5). In order to dissect which cell population is responsible for this higher cytotoxicity, we tested NK and CD8^+^ T cytotoxic cells activity in vitro and found the increased cytotoxicity of both cell types in ST2^−/−^ mice. The ST2 molecule is expressed on human and murine NK cells and IL-33/ST2 signaling may have influence on NK cell function 9, 10. The number of NK cells was decreased in naïve ST2^−/−^ mice, but the total number of IFN-γ-expressing NK cells was markedly higher in ST2^−/−^ mice, before and following tumor challenge (Fig. 4). On the contrary, the number of IL-10-producing NK cells was higher in WT mice after tumor inoculation.

The role of the IL-33/ST2 axis on NK cells function is not fully understood. There are reports that IL-33 can directly stimulate 9 or indirectly amplify 10 responses of iNKT and NK cells. However, the IL-33-dependent enhancement of IFN-γ production by these cells always required the presence of IL-12. Our results from this breast tumor model appear to be at variance with these reports. It appears that the total number of CD3^−^CD19^−^NKp46^+^ cells in the spleen is higher in WT than in ST2^−/−^ mice prior to tumor inoculation (Fig. 4A) and decreases after tumor challenge.

Furthermore, our results suggest that at least during in vivo tumorigenesis ST2 deletion favors the induction of IFN-γ-producing NK cells (Fig. 4B). Additionally, NK cell-mediated cytotoxicity was higher in ST2^−/−^ mice prior to and following tumor challenge (Fig. 5A). One possible explanation of this discrepancy may be related to in vivo maturation of dendritic cells in ST2^−/−^ mice and their effect on NK cells 39. Dendritic cells with a mature phenotype appear to be required for the functional maturation of NK cells 39. Mayuzumi et al. 13 have recently demonstrated that conventional myeloid DCs from IL-33 supplemented cultures are immature and resistant to phenotypic and functional maturation. Thus, it could be assumed that an in vivo lack of ST2 signaling may facilitate maturation of dendritic cells. In fact, we have obtained data indicating that percentage and number of CD11c^+^CD80^high^CD86^high^ DCs was significantly higher in the local lymph nodes of ST2^−/−^ tumor-bearing mice compared with WT mice (data not shown).

We next analyzed NK cells for their constitutive functional phenotype. In mice, CD27 expression divides NK cells into CD27^high^ and CD27^low^ subpopulations with distinct responsiveness and migratory capacity 40. CD27^high^ NK cells showed greater responsiveness to activating ligands expressed on tumor cells and demonstrated effective cytotoxicity against tumor target cells 40. The C-type lectin receptor CD69 represents an early activating marker and may play a direct role in mediating cytotoxicity against tumor target cells 41, whereas KLRG1 is a C-type lectin inhibitory receptor, expressed on a subset of mature NK cells that produce low levels of IFN-γ and have a slow in vivo turnover rate and low proliferative responsiveness to IL-15 42. ST2^−/−^ mice have a significantly higher percentage of CD27^high^CD11b^high^ NK cells as well as immature CD27^high^ CD11b^low^ NK cells, but a lower percentage of CD27^low^ CD11b^high^ NK cells. Additionally, ST2^−/−^ mice have higher percentages of CD69^+^ and KLRG^−^ NK cells. Thus, our data suggest that ST2^−/−^ mice have a significantly higher percentage of highly active, cytokine producing, cytotoxic, and tumoricidal NK cells and immature NK cells (Fig. 6), suggesting that ST2^−/−^ mice may have faster turnover of NK cells and enhanced cytotoxic activity when compared with WT mice.

Finally, in order to analyze how the data of in vitro-enhanced cytotoxicity of CD8^+^ and NK cells in ST2^−/−^ mice relate to in vivo tumor growth, we performed experiments of in vivo depletion of either CD8^+^ or NK cells. In CD8-depleted mice, tumor growth remained slower in ST2^−/−^ mice, whereas in NK-depleted mice the difference in tumor growth between ST2^−/−^ and WT mice could no longer be observed. This finding indicates that NK cells play a more important role than CD8^+^ cells in the enhanced antitumor immunity in ST2^−/−^ mice.

In summing up, we provide the first evidence that deletion of ST2 signaling may enhance antitumor immune response in a murine model of metastatic breast carcinoma. The increased antitumor immunity of ST2-deficient mice could be based on two independent mechanisms, both directed against tumor cells: Th1/Th17 polarization of adaptive immune response with increased proinflammatory cytokines and cytotoxic activity of NK cells. In addition, these two mechanisms could interact and complement each other. Thus, it appears that IL-33/ST2 signaling facilitates primary tumor progression and metastatic dissemination probably by affecting the cytotoxic activity and cellular makeup of local lymph nodes and spleen, indicating an important regulatory role of the IL-33/ST2 pathway in NK physiology and antitumor immunity.

## Materials and methods

### Mice

Female BALB/c (WT) and ST2 knockout (ST2^−/−^) mice on BALB/c background (generated as described previously by Townsend et al. 28), 10 to 11 wk old, were used in experiments. ST2^−/−^ and WT mice were housed under standard laboratory conditions. The WT and the ST2^−/−^ mice were kept in two locations (Kragujevac, Serbia and Rijeka, Croatia) and they were intercrossed and typed. The experiments were approved by the Animal Ethics Board of the Faculty of Medicine, University of Kragujevac, Serbia.

### 4T1 tumor cell line

The weakly immunogenic mouse breast tumor cell line 4T1 that is syngeneic to the BALB/c background was purchased from the American Type Culture Collection (ATCC, USA). 4T1 cells were maintained in Dulbecco's-Modified Eagles Medium supplemented with 10% fetal bovine serum, 2 mmol/L l-glutamine, 1 mmol/L penicillin–streptomycin, 1 mmol/L mixed nonessential amino acids (Sigma, USA) (complete growth medium). Subconfluent monolayers, in log growth phase, were harvested by brief treatment with 0.25% trypsin and 0.02% EDTA in phosphate-buffered saline (PBS, PAA Laboratories GmbH) and washed three times in serum-free PBS before use in all in vitro and in vivo experiments. The number of viable tumor cells was determined by trypan blue exclusion and only cell suspensions with ≥95% viable cells were used.

### Estimation of in vivo tumor growth and progression in 4T1 metastatic breast cancer model

Mice were injected with 50 μL of single-cell suspension containing 5×10^4^ 4T1 mammary carcinoma cells orthotopically into the fourth mammary fat pad of mice as described previously 43. The size of primary tumors was assessed morphometrically from day 7 using electronic calipers in two dimensions. Tumor volumes were calculated according to the formula: tumor volume (mm^3^)=*L*×*W*^2^/2, where *L* represents the major axis (largest cross-sectional diameter) of the tumor, whereas *W* represents minor axis 44, and the data were presented as mean+standard error (mean+SEM). Thirty-six days after tumor cell injection, mice were sacrificed and the primary tumors were surgically removed. Blood was taken from the abdominal aorta. Lung, liver, and brain tissue were collected for the assessment of the presence of metastasis. Specimens of lungs, liver, and brain tissue were routinely embedded in paraffin, stained with hematoxylin and eosin (H&E) and reviewed to confirm the presence of metastatic colonies. Although tumor cells appeared heterogeneous in size, they were easily differentiated as predominately larger cells with an elevated nuclear to cytoplasm ratio. In total, 4 μm hematoxylin and eosin-stained sections from at least three different levels were examined. The number and size of metastatic colonies was examined with light microscope by an independent observer.

### Measurement of cytokines

Serum levels of IL-17, IFN-γ, IL-4, and TNF-α were measured using highly sensitive enzyme-linked immunosorbent assay (hs-ELISA) kits (R&D Systems, Minneapolis, MN, USA), specific for the mouse cytokines, according to the manufacturer's instructions.

### Cellular analysis of local lymph nodes and spleens

Thirteen days after 4T1 tumor cells injection, mice were sacrificed and single-cell suspensions from spleen and the local lymph node (the nearest lymph node that drains the primary tumor) were obtained by mechanical dispersion through cell strainers (BD Pharmingen, USA) in complete growth medium. Additionally, erythrocytes from spleen were removed using lysing solution (BD Pharmingen). After three washes, cells were resuspended in complete growth medium.

### Flow cytometry

Single-cell suspensions of local lymph nodes and spleens were incubated with mAbs specific for mouse CD3, CD4, CD8, CD19, F4/80, NKp46, CD107a, NKG2D, CD27, CD11b, CD69, and KLRG1 or isotype-matched controls (BD Pharmingen/eBioscience) and analyzed by FACSAria flow cytometer (BD Biosciences). Dead cells were excluded by gating out propidium iodide-positive cells. The gate used for FACS analysis is the mononuclear cells region in FSC/SSC plot. Data were analyzed using CELLQUEST software and DiVa (BD).

### Intracellular cytokine staining

Single-cell suspensions of spleens were stimulated with Phorbol 12-myristate 13-acetate (50 ng/mL) (Sigma) and Ionomycin (Sigma) (500 ng/mL) with GolgiStop (BD Pharmingen), and incubated 4 h at 37°C, 5% CO_2_. Cells were stained with anti-NKp46 mAb or isotype-matched control. Subsequently, cells were fixed, permeabilized, and intracellular staining was done using anti-IFN-γ and anti-IL-10 mAb (BD Pharmingen) and analyzed by flow cytometry.

### NK cell separation

NK cells were isolated from spleen and local lymph node by magnetic cell sorting. Target cells were labeled using FlowComp™ Mouse CD49b Ab (Invitrogen, USA), captured by the Dynabeads (Invitrogen), and positively selected using magnet (Invitrogen). In the last step, the beads were removed from the cells, using FlowComp™ Release buffer (Invitrogen).

### CD8^+^ T-cell separation

CD8^+^ T cells were isolated from spleen and the local lymph node by depleting non-CD8^+^ T cells (CD4^+^ T cells, B cells, monocytes/macrophages, NK cells, DCs, erythrocytes, and granulocytes). Mixture of monoclonal Abs against non-CD8^+^ T cells (Invitrogen) was added to cell suspensions, following by Mouse Depletion Dynabeads (Invitrogen) and bead-bound cells were separated by a magnet (Invitrogen). Remaining cells were highly enriched mouse CD8^+^ T cells (purity, >90%).

### Cytotoxicity assays

Cytotoxic activity of lymph node cells, splenocytes, enriched NK cells, enriched CD8^+^ T cells, and adherent cells was measured using the 4 h MTT (3-(4,5-dimethylthiazol-2-yl)-2,5-diphenyltetrazolium bromide, Sigma) assay at various target–effector (*T*:*E*) ratios as described previously 41. 4T1 mouse breast tumor cells were used as targets. The percentage of cytotoxity was calculated as: cytotoxity (%)=(1−(experimental group (OD)/control group (OD)))×100. Data were expressed as the mean of triplicate wells+SEM. Cytotoxic activity was also presented by lytic units, LU_20_/10^7^ cells, calculated from means of triplicate percentages of killing obtained in four different *T*:*E* ratios 45.

### In vivo depletion of NK and CD8^+^ cells during tumor challenge

For in vivo depletion of NK and CD8^+^ cells, mice were treated i.p. with 100 μg of anti-CD8 mAb (YTS 169.4) or with 20 μL of anti-asialo GM1 (Waco. Bioproducts), 1 day prior to and 5 days after tumor inoculation. The efficacy of depletion of cells was assessed 24 h after Ab administration by flow cytometry of splenocytes stained with APC-labeled anti-CD8 and APC-labeled NKp46 Abs (eBioscience). The level of depletion was >95% for both cell subsets.

### Statistical analysis

The data were analyzed using statistical package SPSS, version 13. The two-tailed Student's *t*-test or nonparametric Mann–Whitney Rank Sum test were used. The normality of distribution was tested by Kolmogorov–Smirnov test. The results were considered significantly different when *p*<0.05 and highly significantly different when *p*<0.01.

## Abbreviations

KLRG1: killer cell lectin-like receptor G1
*T:E*: target–effector
